# Peak Oxygen Uptake is Slope Dependent: Insights from Ground Reaction Forces and Muscle Oxygenation in Trained Male Runners

**DOI:** 10.1186/s40798-024-00746-0

**Published:** 2024-07-12

**Authors:** Marcel Lemire, Frédéric Meyer, Rosalie Triguera, Fabrice Favret, Grégoire P. Millet, Stéphane P. Dufour

**Affiliations:** 1 Faculty of Sport Sciences, University of Strasbourg, Strasbourg, France; 2 Institute of Sport Sciences UNIL, University of Lausanne, 1915 Lausanne, Switzerland; 3 Department of Informatics, Digital Signal Processing Group, University of Oslo, Oslo, Norway; 4 Faculty of Medicine, Translational Medicine Federation (FMTS), University of Strasbourg, UR 3072, CEERIPE, Strasbourg, France

**Keywords:** Biomechanics, Cardiorespiratory responses, Tissue saturation index, Eccentric muscle action, Graded running, Muscle fatigue, Performance

## Abstract

**Background:**

The aim of this study is to explore the effect of treadmill slope on ground reaction forces and local muscle oxygenation as putative limiting factors of peak oxygen uptake in graded maximal incremental running tests. Thirteen trained male runners completed five maximal incremental running tests on treadmill at − 15%, − 7.5%, 0%, 7.5% and 15% slopes while cardiorespiratory and local muscle oxygenation responses as well as ground reaction forces were continuously recorded. Blood lactate concentration and isometric knee extensor torque were measured before and after each test.

**Results:**

Peak oxygen uptake was lower at − 15% slope compared to all other conditions (from − 10 to − 17% lower, *p* < 0.001), with no difference between − 7.5 and + 15% slope. Maximal heart rate and ventilation values were reached in all conditions. The negative external mechanical work increased from steep uphill to steep downhill slopes (from 6 to 92% of total external work) but was not correlated with the peak oxygen uptake reduction. Local muscle oxygenation remained higher in − 15% slope compared to level running (*p* = 0.003).

**Conclusions:**

Similar peak oxygen uptake can be reached in downhill running up to − 7.5% slope. At more severe downhill slopes (i.e., − 15%), greater negative muscle work and limited local muscle deoxygenation occurred, even in subjects familiarized to downhill running, presumably preventing the achievement of similar to other condition’s peak oxygen uptake.

**Key Points:**

- Trained male runners can reach like level running V̇O_2peak_ at moderate but not at severe negative slope.
- Negative external mechanical work increases with increasing negative slope.
- At maximal intensity Vastus Lateralis muscle oxygenation is higher in steep negative slope.
- Knee extensor isometric muscle torque is preserved after maximal level and uphill running, but reduced after downhill running, despite lower blood lactate.
- Progressive reduction of V̇O_2_ at maximal effort with increasing negative slope might be related to the metabolic consequences of increased lower limb negative external work (i.e., eccentric muscle actions).

## Background

Experimental evidences emphasize that downhill running (DR) requires lower limb braking muscle actions and is considered mainly as an eccentric exercise modality [1]. By contrast, uphill running (UR) preferentially requires propulsive concentric muscle contractions [1]. Whereas the human’s physiological responses to graded terrain are currently extensively investigated, the key performance factors of DR vs UR might differ, with a greater contribution of biomechanical parameters to DR performance [2]. Moreover, the possibility to achieve high running velocities despite reduced metabolic and cardiorespiratory loads [3] makes DR a promising exercise modality both in athletic and clinical settings [4].

Maximal incremental running tests recently revealed that peak oxygen consumption (V̇O_2peak_) in a homogenous group of well-trained runners is 16–18% lower during DR on a steep slope (DRSS, − 15% slope) than during level (LR, 0% slope) or uphill running on a steep slope (URSS, + 15% slope) [3]. However, the possibility that V̇O_2peak_ could still be achieved at less severe slopes in DR (i.e., moderate slope, DRMS) remains an open question.

The achievement of V̇O_2peak_ in DR, even at moderate slope, ineluctably requires reaching high to very high running velocities. Although modern treadmills allow such specific high intensity efforts to be performed in a secured setting, very limited information is currently available for characterizing the physiological, muscle fatigue and especially mechanical components of maximal DR exercise. So far, only two studies have combined physiological and biomechanical explorations in DR, albeit restricted to low velocity [5, 6], particularly highlighting that stride spatiotemporal parameters are self-adjusted to induce the lowest oxygen cost of running at 10 km·h^−1^ on moderate slopes (i.e., >  − 10%). Therefore, the potential relationship between ground reaction forces and cardiorespiratory responses in DR has never been investigated, yet the greater magnitude of, for instance, braking forces (i.e., eccentric muscle actions) could contribute to the lower V̇O_2peak_ observed in DRSS.

Eccentric muscular actions can be quantified from the negative values of the parallel and normal components of the ground reaction during foot contact, which correspond to the phase of braking and absorption of mechanical energy [7, 8]. Available data suggest the steeper the negative slope, the greater the braking forces at a given speed (~ 11 km·h^−1^) with the highest values in braking forces [9] and negative mechanical external work (at 8 to 20 km·h^−1^) [7] occurring at slopes of − 15%. How the magnitude of negative external mechanical work actually influences cardiorespiratory responses and potentially prevents the achievement of maximal oxygen consumption (V̇O_2max_) in DR remains an open question.

In addition to mechanical parameters, restriction of muscle deoxygenation might also be involved in the limitation of V̇O_2max_ in DR. Indeed, an increase in tissue oxygen extraction is commonly assumed to reflect an enhancement in the body’s ability to utilize oxygen during exercise, which may contribute to an increases V̇O_2peak_, and vice versa. During incremental exercise to exhaustion, either in level running or cycling, oxygen consumption (V̇O_2_) increases linearly to meet the increasing metabolic demand until V̇O_2max_ is attained [10], while deoxygenated haemoglobin ([HHb]) signal derived from near-infrared spectroscopy (NIRS) in *Vastus Lateralis* muscle shows a linear increase to a point where a plateau-like response occurs before V̇O_2max_ is reached [11]. However, the muscle oxygenation status of locomotor muscle during maximal DRSS remains unknown to date.

Therefore, the primary aim of this study was to determine the effect of moderate and steep treadmill slopes on running biomechanics, cardiorespiratory responses, and local muscle oxygenation achieved at exhaustion during maximal incremental running exercise. A secondary aim of this investigation was to determine whether oxygen uptake achieved at maximal intensity is related to slope-dependent responses in running biomechanics and/or local muscle oxygenation.

## Methods

### Experimental Design

A group of thirteen trained male runners volunteered to take part in this study (age: 25 ± 2 [mean ± SD] years; height: 1.78 ± 0.05 m; body mass: 72.7 ± 4.9 kg; LR V̇O_2peak_: 60.1 ± 4.1 ml·min^−1^·kg^−1^; LR velocity at V̇O_2peak_: 16.4 ± 1.2 km·h^−1^). The subjects included in this study had to meet the following criteria: be a trained runner with a V̇O_2max_ greater than or equal to 50 mlO_2_·kg^−1^·min^−1^, male, non-smoker or not having smoked for the past 5 years, aged between 18 and 45 years, with a body mass index less than 25, affiliated with a health insurance system, and having signed an informed consent form. Subjects were excluded from the study if they had any of the following characteristics: inability to provide informed consent (emergency situation, comprehension difficulties, etc.), under legal protection, guardianship, or curatorship, contraindications to physical and sports activities, musculoskeletal or joint problems of the lower limbs, respiratory, cardiovascular, or metabolic pathology, or ongoing medication that could not be stopped within 7 days before the first visit. Based on the Participant Classification Framework using training volume and performance metrics, the participants are classified as Tier 2: Trained/Developmental [12]. The participants were primarily recruited from the population of well-trained male runners engaged in aerobic running activities, registered in sports clubs in Lausanne (Switzerland). Communication about this study was carried out by the associated researchers, as well as through small advertisements and postings in targeted locations (clubs and associations). All participants were informed of the benefits and risks of this investigation prior to giving their written informed consent to participate. The experiment was previously approved by the local ethical committee (CCER-VD 2015-00006; Commission cantonale d’éthique de la recherche sur l’être humain. Canton de Vaud, Switzerland) and complied with the Declaration of Helsinki. All participants visited the laboratory on nine occasions in order to perform each of the experimental tests: i) a level running (0% slope) incremental test to exhaustion, ii) four DR familiarization sessions, iii) four randomized inclined running incremental tests to exhaustion (± 7.5% and ± 15% slopes). Each session was performed at the same time of day and separated by at least 7 days of recovery.

### Maximal Incremental Running Tests in Level and Downhill Conditions

After the four DR familiarization sessions at progressive intensity and duration increases, all participants performed the five incremental tests until exhaustion on an instrumented treadmill (T-170-FMT, Arsalis, Belgique). At the first session, the participants performed the LR test: the first stage began at 7.5 km·h^−1^ and the running speed increased by 1.2 km·h^−1^ at each stage. During the DRSS trial (− 15% slope), the first stage began at 7.5 km·h^−1^ and the running speed increased by 1.7 km·h^−1^ at each stage. During the DRMS (− 7.5% slope), the first stage began at 7.5 km·h^−1^ and the running speed increased by 1.5 km·h^−1^ at each stage. During the UR on moderate slope (URMS, + 7.5% slope), the first stage began at 4.5 km·h^−1^ and the running speed increased by 1 km·h^−1^ at each stage. During the URSS trial (15% slope), the first stage began at 4.5 km·h^−1^ and the running speed increased by 0.5 km·h^−1^ at each stage. These protocols were defined to obtain similar effort times based on preliminary tests. During all tests, stage duration was set at 2-min. One-min rest-periods with the subjects standing on the treadmill were used to record baseline values before each test. These protocols were established from pilot studies to provide a similar time to exhaustion across the five conditions. During each incremental running test, oxygen uptake (V̇O_2_), carbon dioxide output, ventilation, breathing frequency, tidal volume, and respiratory exchange ratio were collected breath-by-breath though a facemask with an open-circuit metabolic cart with rapid O_2_ and CO_2_ analyzers (Quark CPET, Cosmed, Rome, Italy). Before each exercise test, the pneumotachograph as well as the O_2_ and CO_2_ analyzers were calibrated according to manufacturer’s instructions. Heart rate (HR) was continuously measured (Polar Electro, Kempele, Finland). V̇O_2max_ was defined as the highest 30 s V̇O_2_ value in each test. The velocity associated with V̇O_2max_ was determined as the minimal velocity associated to V̇O_2max_ [13]. For clarity and because V̇O_2max_ was not reached in DRSS, we used the terminology “peak” values to refer to the maximal values achieved for a given parameter in each experimental condition [3, 14, 15]. Mass-related V̇O_2peak_ was used for the analysis, except when absolute value was mentioned. Peak O_2_ pulse was calculated by dividing V̇O_2peak_ with HR peak values. Thirty seconds after the end of the test, the participants were asked to rate their perceived exertion (RPE) to assess the global intensity of the test as well as the levels of breathing and muscle exertion [16].

### Blood Lactate Analyses

Blood lactate concentration (*b*[La]^−^) was assessed from finger blood samples (50 µl of blood) before and after 1 and 3 min of recovery after each maximal incremental running test using an enzymatic amperometric detection method (Lactate Scout + , EKF Diagnostics, Leipzig, Germany).

### Near-Infrared Spectroscopy

Oxyhaemoglobin ([O_2_Hb]), deoxyhaemoglobin [HHb] and total haemoglobin ([tHb] = [O_2_Hb] + [HHb]) concentrations and tissue saturation index [(TSI (%) = (O_2_Hb/tHb) × 100] of the *Vastus Lateralis* of the dominant leg were measured by NIRS (PortaLite device and Oxysoft software, Artinis Medical Systems, Elst, The Netherlands). This muscle has been shown to be markedly used in UR and DR [17]. The transmitting and receiving optodes were positioned with 35 mm interoptode spacing, on the lower third between the lateral epicondyle and greater trochanter of the femur, parallel to the long axis of the muscle. To enable reproducible probe placement between the sessions, the location was marked with indelible ink. The optode assembly was secured on the skin surface with tape and then covered with an optically dense, black tissue sleeve to minimize the intrusion of extraneous light. The thigh was wrapped with an elastic bandage to minimize optode movement. Light absorption at different wavelengths (from 750 to 850 nm) was sampled at a frequency of 50 Hz and analyzed according to the modified Beer-Lambert law. A second order stop-band Butterworth filter with frequency set to 15 Hz was applied and a differential pathlength factor of 4 was used.

To provide equivalence for both [O_2_Hb] and [HHb] signal sensitivity (i.e., allowing for minor differences arising from differences in optode placement and/or day-to-day variability in signal intensity), [O_2_Hb] and [HHb] profiles were normalized from the value measured during the rest period just before each maximal incremental test [18].

This procedure does not affect the TSI value because TSI is measured as an absolute value instead of a change with respect to the arbitrary initial baseline value. Then normalized [O_2_Hb], [HHb], and TSI values were averaged during the last 30 s of each stage.

A normalized Δ[HHb]:ΔV̇O_2_ ratio derived from each subject was averaged over 30 s for resting and peak values [19]

### Biomechanical Data Collection and Processing

Data from the integrated three-dimensional force platform were sampled at 1000 Hz. To reduce the noise inherent to the treadmill’s vibrations, we first applied, a second order stop-band Butterworth filter with edge frequencies set at 25 and 65 Hz, on the vertical ground reaction force signal. The filter configuration was chosen empirically to obtain a satisfactory reduction of the oscillations observed during flight phases while minimizing its widening effect during ground contact time. Further data analysis was conducted using MATLAB software version R2021a (MathWorks Inc., Natick, MA, USA).

The instants of initial contact and terminal contact were identified using a threshold of 7% of bodyweight on the filtered vertical ground reaction force signal [20]. The contact time (in ms) is the time between initial and terminal contacts of the same leg, the flight time (in ms) is the time between the terminal contact of one leg and the initial contact of the opposite leg. The step frequency (in Hz) is the reciprocal of the time required for one step (time between two consecutive initial contacts). The step length (m) is the quotient of the treadmill belt speed divided by step frequency. Peak ground reaction force and impulse in the normal direction were computed over the entire stance phase. The algorithm determined the normal impact peak force magnitude by starting from foot strike and finding the value when the positive slope of the normal force versus time became negative. The normal active peak force was determined by starting from toe-off and working backwards in time towards foot strike. This procedure was also applied to determine parallel braking and propulsive peak forces; both being separated by the instant the negative parallel force becomes positive during midstance. We obtained normal and parallel impulse data by integrating all the positive (normal and parallel propulsive impulse) or negative (parallel braking impulse) values of the ground reaction forces during each ground contact. Peak normal and parallel components of the ground reaction force as well as impulse data were obtained during each stance phase and averaged over 30 s, considering the treadmill inclination. The external mechanical work (W_ext_) as determined using the method developed by Cavagna [21] and internal mechanical works (W_int_) was calculated using the equations proposed by Minetti [22]. The percentage of negative work is the ratio between the negative external work (W_ext_^−^) and the total external work. The total lateral force is the sum of the of the two absolute values of the peak lateral forces. These data were continuously recorded during 30 s between 1:00 and 1:30 (min:s) of each stage.

### Lower Limb Muscle Fatigue Assessment

Before each maximal incremental running test and after 1 and 3 min of recovery, each subject performed maximal isometric voluntary contractions of the knee extensor muscles. These tests were performed on a custom-built chair ergometer equipped with a force gauge (Universal Load Cell, VPG Revere transducers, Germany) at the ankle. Participants sat with a 90° hip angle; the right knee positioned at 90° of flexion (0° = fully extended). The lever arm was attached 2 cm above the internal malleolus with a non-compliant strap. To avoid upper body movement, participants were attached on the trunk with non-compliant straps and arms were crossed on their chest. Strain-gauge data were recorded using the Biopac MP150 system (Biopac System, Santa Barbara, CA) with an acquisition frequency of 1000 Hz and stored for analysis with dedicated software (AcqKnowledge 4.2 for MP systems, Biopac System, Santa Barbara, CA). To ensure reliability of the measurements across sessions, the ergometer’s participant settings (i.e., lever arm length and chair adjustments) were kept constant between sessions. For the maximal voluntary contraction performed at the beginning of each incremental test, the participant had first to perform three 5 s submaximal voluntary contractions, with 30 s rest in-between, to warmup. Then, two 5 s maximal efforts were performed with strong verbal encouragements, with 30 s rest in-between. A third one was performed if the second was better than the first one and the highest score reached was retained. For the post-exhaustion force evaluation, two 5 s maximum voluntary contractions were performed with 30 s rest in-between, after the end of the test. These measurements were initially collected in Newton and subsequently converted in torque using anatomic measures of knee extensor lever arms. Lower limb muscle fatigue was assessed using the relative force decrement observed between pre and post exercise values. All measurements were performed by two experienced investigators.

Delayed onset muscle soreness (DOMS) was assessed 24, 48 and 72 h after each maximal incremental test, using a scale of perceived soreness (0 for no soreness to 10 for maximal soreness) for each of the following muscle groups: hip extensors, knee extensors, knee flexors, plantar flexors and plantar extensors.

### Statistical Analysis

Statistical analyses were performed using Statistica (13.5, Tulsa, Oklahoma, USA). All data are expressed as mean ± standard deviation (SD). The normality of the distribution was assessed for all variables using the Shapiro–Wilk test. For the non-normal variables, data were log-corrected by applying the Box-Cox method to ensure appropriate use of the ANOVA. Sphericity was checked with the Mauchly’s test and a Greenhouse–Geisser correction to the degree of freedom was applied when sphericity was violated. One-way ANOVAs on repeated measures were performed to assess the effect of slope on cardiorespiratory, metabolic, cardiovascular, neuromuscular and biomechanical parameters obtained at maximal effort. When significant effects were observed, Tukey’s post-hoc tests were used to localize the significant differences. Pearson’s product moment correlation coefficients (*r*) were used to assess possible relationships between variables. For all analyses, *p* < 0.05 was considered statistically significant.

## Results

### Oxygen Uptake, Running Velocities and Blood Lactate Responses at Maximal Intensity

**Fig. 1:**
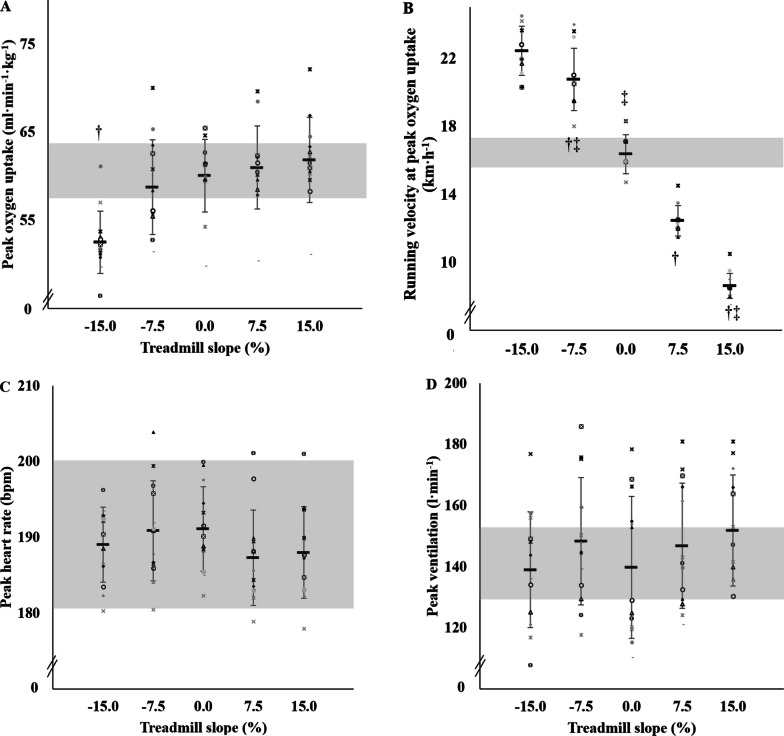
Peak oxygen uptake (Panel **A**), running speed at Peak oxygen uptake (Panel **B**), peak heart rate (Panel **C**) and maximal ventilation (Panel **D**) as function of treadmill slope. For each panel, individual data, and the average (bold line) and ± SD of 13 participants are shown. The grey area corresponds to the 5% deviation from the mean value as confidence interval.  subject 1,  subject 2,  subject 3,  subject 4,  subject 5,  subject 6,  subject 7,  subject 8,  subject 9,  subject 10,  subject 11,  subject 12,  subject 13. † *p* < 0.05 versus level running (i.e., LR, 0% slope). ‡ *p* < 0.05 vs. steep slope downhill running (i.e., DRSS, − 15% slope)

Peak oxygen uptake was similar across all conditions (all *p* > 0.138) except for DRSS which exhibited a lower value (range: − 10 to − 17% vs. all other conditions, *p* < 0.001; Fig. 1A). Compared to LR vV̇O_2peak,_ DRSS vV̇O_2peak_ was 38 ± 12% higher, DRMS vV̇O_2peak_ was 27 ± 9% higher, URMS vV̇O_2peak_ was 24 ± 4% lower and URSS vV̇O_2peak_ was 47 ± 3% lower (Fig. 1B). Peak O_2_ pulse was different in-between all conditions (*p* < 0.05), except between LR and DRMS. One and 3-min post-exercise blood lactate concentrations were similar between all conditions (all *p* ≥ 0.272), except for DRSS where post 1 and 3-min values were lower compared to all other conditions (range: − 25 to − 33%; all *p* ≤ 0.032, Table 1).

**Table 1:** Oxygen uptake, respiratory and blood lactate responses at maximal intensity in downhill, level and uphill running

Treadmill slope	− 15%	− 7.5%	0%	+ 7.5%	+ 15%
Peak heart rate (bpm)	189 ± 5	191 ± 7	191 ± 6	187 ± 6	188 ± 6
Peak oxygen uptake (ml·min^−1^)	3869 ± 288^†^	4324 ± 496^‡^	4364 ± 353	4500 ± 359^‡^	4557 ± 423^‡^
Peak carbon dioxide output (ml·min^−1^)	3747 ± 363^†^	4311 ± 506^‡^	4344 ± 501	4528 ± 366^‡^	4511 ± 480^‡^
Peak respiratory exchange ratio	0.97 ± 0.05	1.00 ± 0.06	0.99 ± 0.05	1.01 ± 0.06	0.99 ± 0.06
Peak O_2_ pulse (ml·beats^−1^)	20.5 ± 1.7^†^	22.7 ± 2.6^†,‡^	22.8 ± 1.7	24.1 ± 2.2^†,‡^	24.2 ± 2.2^†,‡^
Running speed at V̇O_2peak_ (km·h^−1^)	22.4 ± 1.5^†^	20.8 ± 1.8^†,‡^	16.4 ± 1.2	12.4 ± 0.9^†,‡^	8.6 ± 0.7^†,‡^
Running speed at exhaustion (km·h^−1^)	22.5 ± 1.5^†^	20.9 ± 1.7^†,‡^	17.1 ± 0.8	12.8 ± 1^†,‡^	8.8 ± 0.8^†,‡^
Exhaustion time (min:sec)	19′00 ± 1′48	19′36 ± 2′12	18′24 ± 1′36	18′42 ± 1′36	19′06 ± 3′06
Δ[HHb]_peak_/ΔV̇O_2peak_ ratio	3.4 ± 1.7	2.2 ± 1.7	3.7 ± 2.6	3.0 ± 1.6	3.6 ± 1.2
Blood lactate before test (mmol·l^−1^)	1.5 ± 0.3	1.7 ± 0.5	1.9 ± 0.5	1.5 ± 0.4	1.7 ± 0.3
Blood lactate 1 min after test (mmol·l^−1^)	8.3 ± 2.9^†^	11.0 ± 3.0^‡^	11.8 ± 2.3	12.6 ± 2.0^‡^	12.8 ± 2.8^‡^
Blood lactate 3 min after test (mmol·l^−1^)	8.5 ± 4.1^†^	10.1 ± 2.7	12.5 ± 3.2	12.3 ± 2.9^‡^	13.1 ± 4.2^‡^
Breathing frequency (breaths·min^−1^)	64 ± 11^†^	59 ± 7^†^	49 ± 7	51 ± 8^‡^	52 ± 6^‡^
Tidal volume (l)	2.19 ± 0.27^†^	2.52 ± 0.29^†,‡^	2.84 ± 0.24	2.87 ± 0.14^‡^	2.91 ± 0.23^‡^

N = 13. Values are peak ± SD. ΔV̇O_2peak_, normalized peak O_2_ uptake; Δ[HHb]_peak_, normalized peak deoxyhemoglobin; † *p* < 0.05 versus level running (i.e., LR, 0% slope). ‡ *p* < 0.05 vs. downhill running (i.e., DRSS, − 15% slope)

### Cardiorespiratory Responses at Maximal Intensity

Despite significantly different vV̇O_2peak_ (from 53 to 138% of LR vV̇O_2peak_), peak HR (HR_peak_) was reached in each running condition (all *p* ≥ 0.152; Fig. 1C). Participants also attained maximal ventilation in all conditions (Fig. 1D), albeit DRSS and DRMS demonstrated lower peak tidal volumes (− 22 and − 11%, all *p* < 0.001) and greater breathing frequencies (+ 31% and + 21%, *p* < 0.001), when compared to LR (Table 1).

### *Vastus Lateralis* Muscle Oxygenation Saturation

**Fig. 2:**
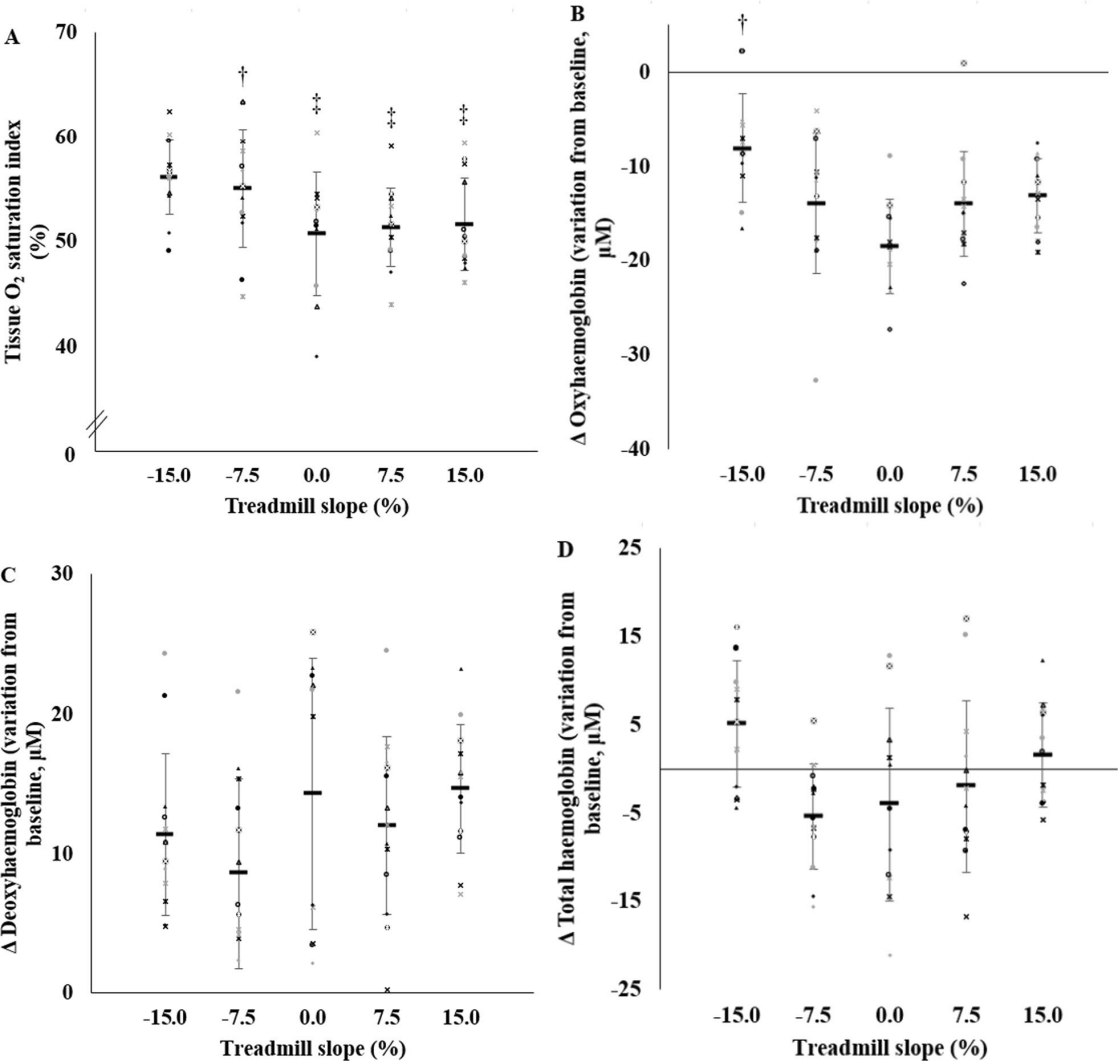
Tissue O_2_ saturation index (Panel **A**), fold oxyhemoglobin variation from baseline (Panel **B**), fold deoxyhemoglobin variation from baseline (Panel **C**) and fold total hemoglobin variation from baseline (Panel **D**) as function of treadmill slope at peak oxygen uptake. For each panel, individual data, and the average (bold line) and ± SD of 13 participants are shown.  subject 1,  subject 2,  subject 3,  subject 4,  subject 5,  subject 6,  subject 7,  subject 8,  subject 9,  subject 10,  subject 11,  subject 12,  subject 13. † *p* < 0.05 versus level running (i.e., LR, 0% slope). ‡ *p* < 0.05 versus steep slope downhill running (i.e., DRSS, − 15% slope)

At maximal intensity, TSI was from 9 to 12% higher in DRSS versus all other conditions (all *p* < 0.021; Fig. 2A), except versus DRMS. In addition, TSI in DRMS was higher than in LR (+ 10%; *p* < 0.016). The Δoxyhaemoglobin, the Δdeoxyhaemoglobin, the Δtotal haemoglobin and the Δ[HHb]:ΔV̇O_2_ ratio values were similar among all conditions, except for Δoxyhaemoglobin in DRSS which was higher than in LR (+ 44%; *p* = 0.002; Fig. 2B–D).

Among muscle oxygenation parameters, only Δdeoxyhaemoglobin and the Δ[HHb]:ΔV̇O_2_ ratio values in DRSS correlated to the difference between LR V̇O_2peak_ and DRSS V̇O_2peak_ (*r* =  − 0.73 and *r* = 0.65, respectively; both *p* < 0.029). Differences in *Vastus Lateralis* muscle oxygenation parameters between DRSS and LR did not correlate with the difference between LR V̇O_2peak_ and DRSS V̇O_2peak_.

### Analysis of Ground Reaction Forces at Maximal Intensity

#### Running Gait Kinematics

At maximal intensity, stride length and frequency increased from URSS to DRSS (all *p* ≤ 0.009; Table 2) with no difference between the two DR conditions. Similarly, the ground contact time shortened, and the aerial time increased from steep uphill to steep downhill slope (all *p* ≤ 0.014) with DRSS and DRMS displaying no significant differences (*p* ≥ 0.752; Table 2).

**Table 2:** Biomechanical parameters measured at maximal intensity in downhill, level and uphill running

Treadmill slope	− 15%	− 7.5%	0%	+ 7.5%	+ 15%
Stride length (m)	2.05 ± 0.16^†^	1.94 ± 0.16^†‡^	1.56 ± 0.14	1.23 ± 0.09^†‡^	0.87 ± 0.08^†‡^
Stride frequency (Hz)	3.02 ± 0.20	3.00 ± 0.22	2.93 ± 0.15	2.89 ± 0.18	2.68 ± 0.23^†‡^
Contact time (s)	0.17 ± 0.02^†^	0.17 ± 0.03^†^	0.21 ± 0.01	0.26 ± 0.02^†‡^	0.30 ± 0.05^†‡^
Aerial time (s)	0.16 ± 0.03^†^	0.16 ± 0.03^†^	0.13 ± 0.02	0.09 ± 0.02^†‡^	0.05 ± 0.03^†‡^
Peak parallel force (N)	214.0 ± 43.0^†^	277.1 ± 57.8^‡^	270.9 ± 34.5	259.5 ± 26.7^‡^	269.5 ± 29.9^‡^
Peak normal force (N)	2448.1 ± 285.2^†^	2120.5 ± 192.6^†‡^	1963.2 ± 183.3	1814.3 ± 161.7^†‡^	1610.9 ± 136.4^†‡^
Total lateral forces (N)	538.5 ± 80.2^†^	421.1 ± 60.1^†‡^	257.6 ± 78.1	257.3 ± 78.1^‡^	323.4 ± 30.8^†‡^
W_ext_ (J·m^−1^·kg^−1^)	3.75 ± 0.75^†^	2.89 ± 0.14^†‡^	2.42 ± 0.15	2.51 ± 0.23^†‡^	3.39 ± 0.23^†‡^
W_ext_^−^/W_ext_ (%)	91.59 ± 1.30^†^	77.31 ± 2.18^†‡^	48.40 ± 0.50	20.58 ± 2.95^†‡^	5.71 ± 3.09^†‡^
Wx_ext_ (J·m^−1^·kg^−1^)	0.055 ± 0.008	0.022 ± 0.003	0.004 ± 0.001	0.030 ± 0.004	0.165 ± 0.183^†‡^
W_int_ (J·m^−1^·kg^−1^)	0.26 ± 0.08	0.27 ± 0.12	0.35 ± 0.09	0.53 ± 0.10^‡^	0.71 ± 0.33^†‡^

N = 13. Values are peak ± SD. W_ext_: external work; Wx_ext_: total lateral work; W_int_: internal work; † *p* < 0.05 versus level running (i.e., LR, 0% slope). ‡ *p* < 0.05 versus downhill running (i.e., DRSS, − 15% slope)

#### Braking Phase

**Fig. 3:**
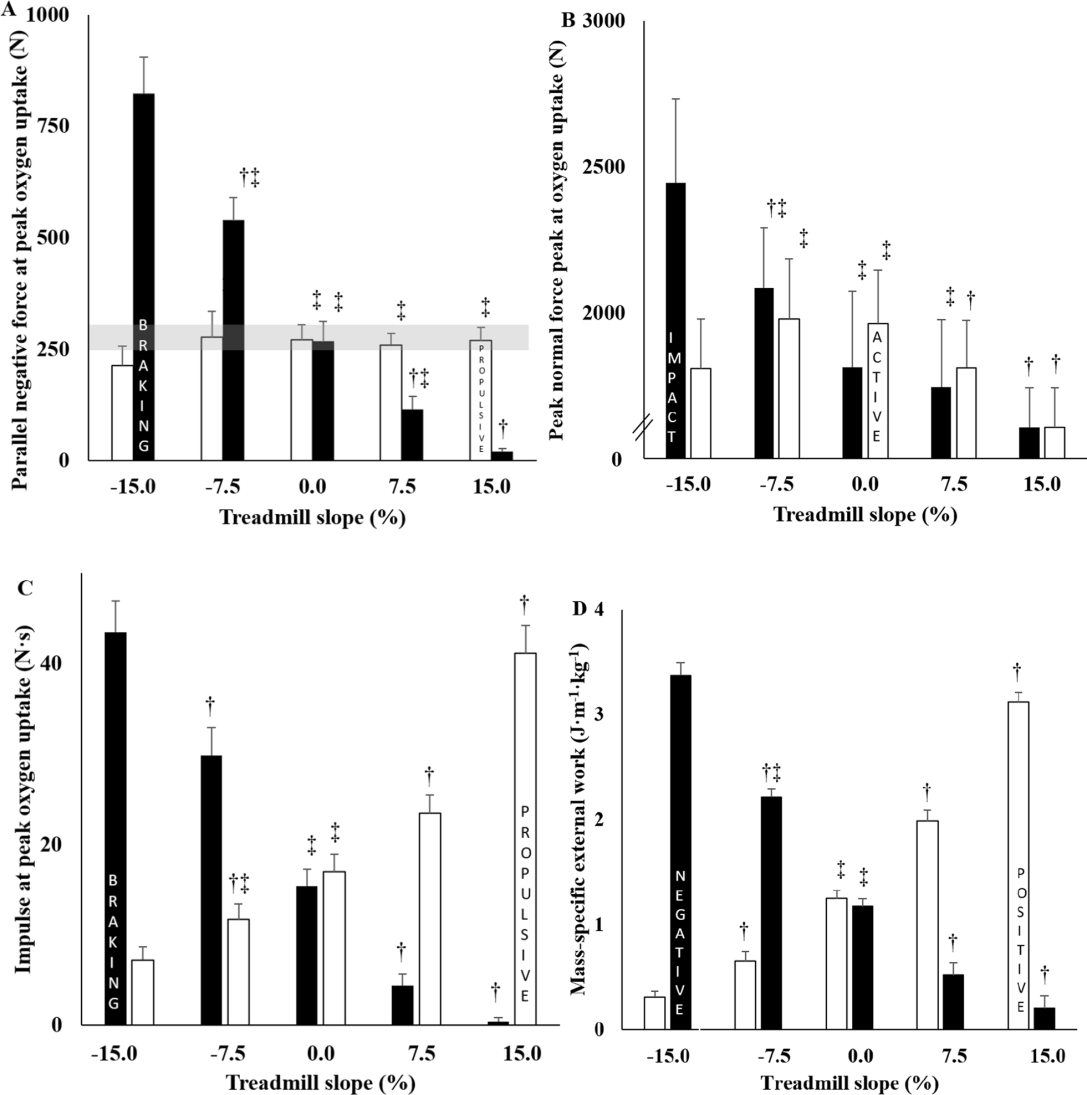
Minimal and maximal parallel forces (Panel **A**), impact and active normal forces (Panel **B**), braking and propulsive impulses (Panel **C**) and negative and positive mass-specific external works (Panel **D**) as function of treadmill slope at peak oxygen uptake. For each panel, mean values ± SD of 13 participants are shown. The grey area corresponds to the 5% deviation from the mean value as confidence interval. † *p* < 0.05 versus level running (i.e., LR, 0% slope). ‡ *p* < 0.05 versus steep slope downhill running (i.e., DRSS, − 15% slope)

The peak values for parallel braking and normal impact forces increased from URSS to DRSS (all *p* < 0.040; Fig. 3A and B), except for the normal impact peak force that remained similar between URMS and LR (*p* = 0.608). The braking impulse increased from URSS to DRSS (all *p* < 0.001; Fig. 3C). The mass-specific negative external mechanical work per unit of distance was three-folds higher in DRSS vs. LR (*p* < 0.001; Fig. 3D). The ratio of negative external work to total external work increased from URSS to DRSS (all *p* < 0.001; Table 2).

#### Propulsive Phase

At vV̇O_2peak_, the peak parallel propulsive ground reaction forces were 17 to 22% lower in DRSS versus all other conditions (all *p* ≤ 0.004; Fig. 3A), with no difference between DRMS through to URSS. The normal active peak force was higher in both LR and DRMS than in all other conditions (all *p* < 0.001; Fig. 3B), and the propulsive impulse decreased from URSS to DRSS (all *p* < 0.001; Fig. 3C). The mass-specific positive external mechanical work per unit of distance was three-quarter lower in DRSS vs. LR (*p* < 0.001; Fig. 3D). The total lateral forces was higher in DRSS versus all other conditions (all *p* < 0.001; Table 2).

### Relationships Between DRSS and LR Responses

Neither biomechanical parameter values, nor differences between LR and DRSS correlated with the difference between LR V̇O_2peak_ and DRSS V̇O_2peak_. Contact time in DRSS was the only spatiotemporal parameter that correlated inversely with the difference between LR V̇O_2peak_ and DRSS V̇O_2peak_ (*r* =  − 0.58; *p* = 0.037). Interestingly, the contact time and the stride frequency differences between LR and DRSS correlated with the difference between LR V̇O_2peak_ and DRSS V̇O_2peak_ (*r* = 0.73; *p* = 0.004 and *r* =  − 0.67; *p* = 0.013, respectively).

The parallel braking forces correlated inversely with V̇O_2peak_ only in DRMS (*r* =  − 0.61; *p* = 0.037). The normal impact peak force correlated positively with V̇O_2peak_ in all conditions (all *r* > 0.63, *p* < 0.022), except in URSS (*r* = 0.36; *p* = 0.225), while the normal active peak force correlated positively with V̇O_2peak_ only in LR and DRMS (both *r* > 0.59; *p* < 0.033). Neither propulsive nor braking impulses correlated with V̇O_2peak_ (all *r* < 0.48, *p* > 0.119), except inversely for braking impulse in DRMS (*r* =  − 0.60; *p* = 0.037).

**Fig. 4:**
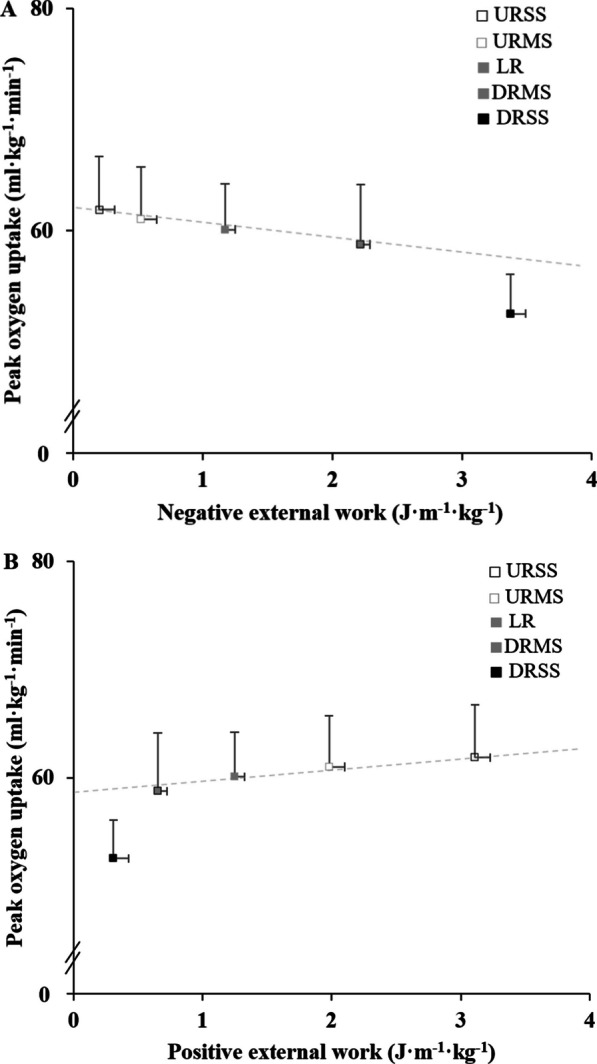
Relationship between peak oxygen uptake and negative and positive external works (Panel **A** and **B**, respectively). DRSS, downhill running in steep slope (− 15%); DRMS, downhill running in moderate slope (− 7.5%); LR, level running (flat); URMS, uphill running in moderate slope (7.5%); URSS, uphill running in steep slope (15%). † *p* < 0.05 versus level running (i.e., LR, 0% slope)

Peak oxygen uptake was not linearly related to the percentage of negative external work, but rather seemed to decrease exponentially, particularly when the percentage of negative external work became greater than ~ 70% (i.e., when DR slope is greater than − 7.5%; Fig. 4 for external work values).

Absolute negative external work in DRSS, DRMS and LR correlated inversely with absolute V̇O_2peak_ in DRSS, DRMS and LR respectively (all *r* >  − 0.62; *p* < 0.039). Positive and negative external works related to body mass and distance were not correlated with body mass-related V̇O_2peak_ in any condition (all *r* < 0.52; *p* > 0.104).

### Global Neuromuscular Fatigue, Delayed-Onset Muscle Soreness and Ratings of Perceived Exertion

No reduction in knee extensor torque was reported after LR, URMS and URSS, whereas a significant torque loss was observed after DRMS and DRSS (*p* < 0.02; Table 3).

**Table 3:** Global neuromuscular fatigue, delayed-onset muscle soreness and rate of perceived effort at peak oxygen uptake in downhill running on steep slope (− 15%), downhill running on moderate slope (− 7.5%), level running (0%), uphill running on moderate slope (+ 7.5%) and uphill running on steep slope (+ 15%)

**Treadmill slope**	**-15%**			**-7.5%**			**0%**	**+7.5%**	**+15%**
Pre absolute knee extensor torque (N·m)	271.1 ± 55.8			262.8 ± 52.2^†^			281.9 ± 60.2	277.9 ± 59.5	268.0 ± 56.3
Post absolute knee extensor torque (N·m)	245.3 ± 52.9^†^			243.7 ± 54.7^†^			266.4 ± 60.9	261.6 ± 60.9^‡^	257.1 ± 56.4^‡^
Post relative knee extensor torque (N·m·kg^-1^)	3.4 ± 0.7^†^			3.4 ± 0.7^†^			3.6 ± 0.7	3.6 ± 0.8^‡^	3.5 ± 0.7
Post torque loss (%)	-11 ± 7^†^			-9 ± 7^†^			-7 ± 11	-1 ± 7^‡^	-5 ± 5^‡^
Global rate of perceived effort	18.5 ± 1.4			19.2 ± 0.7			18.7 ± 2.0	19.5 ± 0.5	19.6 ± 0.5
Muscle rate of perceived effort	19.2 ± 1.4^†^			18.2 ± 2.2			17.6 ± 2.4	18.1 ± 1.6	18.8 ± 1.1
Breathing rate of perceived effort	17.4 ± 1.3^†^			18.8 ± 1.3^‡^			18.6 ± 1.1	19.6 ± 0.7^‡^	19.4 ± 1.0^‡^
	**24-h post**	**48-h post**	**72- h post**	**24-h post**	**48-h post**	**72- h post**	-	-	-
Hip extensor DOMS	4.5 ± 2.1	3.8 ± 2.5	2.2 ± 2.2	2.9 ± 2.5^‡^	2.4 ± 2.1	1.8 ± 2.2			
Knee extensor DOMS	4.7 ± 2.5	4.5 ± 2.5	2.5 ± 1.9	3.4 ± 2.3	3.0 ± 2.4^‡^	2.0 ± 2.6			
Knee flexor DOMS	3.8 ± 2.4	3.6 ± 2.5	2.0 ± 1.9	2.7 ± 2.1	2.5 ± 2.3	1.9 ± 2.2			
Plantar extensor DOMS	2.6 ± 2.5	2.6 ± 2.6	1.8 ± 2.1	2.9 ± 2.6	2.7 ± 2.6	2.1 ± 2.5			
Plantar flexor DOMS	4.8 ± 2.2	4.6 ± 2.7	2.8 ± 2.2	3.2 ± 2.8	2.9 ± 2.9	2.0 ± 2.6			

N = 13. Values are peak ± SD. Delayed onset muscle soreness (DOMS) 24, 48 and 72-h post exercise. † *p* < 0.05 versus level running (i.e., LR, 0% slope). ‡ *p* < 0.05 versus downhill running (i.e., DRSS, − 15% slope)

The global RPE at exhaustion was similar in all conditions (Table 3). The muscle RPE at exhaustion was 10% higher only in DRSS compared to LR (*p* = 0.010), whereas the breathing RPE was lower (from − 6 to − 13%) only in DRSS versus all other conditions (*p* ≤ 0.030).

Delayed onset muscle soreness occurred at 24 h post exercise only after DRSS and DRMS for hip extensor, knee extensor, knee flexor, and plantar flexor muscles and remained elevated at 48 and 72 h.

All vV̇O_2peak_ were correlated together (all 0.65 ≤ *r* ≤ 0.95; *p* ≤ 0.015), except for DRSS vV̇O_2peak_ which only tended to correlate with all other conditions (all 0.51 < *r* < 0.55; *p* < 0.1) but not with LR vV̇O_2peak_ (*r* = 0.26; *p* = 0.384).

The torque losses did not correlate with vV̇O_2peak_ in DRMS or DRSS (both *r* < 0.08; *p* > 0.788).

Pre and post knee extensor torques were positively correlated to vV̇O_2peak_ in DRSS (*r* > 0.58; both *p* < 0.036; Table 3). All pre and post knee extensor torques were positively correlated or tended to be positively correlated with vV̇O_2peak_ (all *r* > 0.50;* p* < 0.081) and V̇O_2peak_ (all *r* > 0.51; *p* < 0.078). The difference between LR V̇O_2peak_ and DRSS V̇O_2peak_ did not correlate to knee extension torque nor with DRSS knee extension torque losses (all *p* > 0.394).

None of the DOMS values correlated with DRSS/DRMS V̇O_2peak_ nor with the difference between LR V̇O_2peak_ and DRSS/DRMS V̇O_2peak_.

## Discussion

This study is the first comprehensive analysis of the effect of moderate and steep slopes on the cardiorespiratory responses, running mechanical characteristics and local muscle oxygenation responses to maximal intensity running in trained male runners. Our results demonstrate that: i) trained male runners can reach LR V̇O_2peak_ at moderate but not at severe negative slope; ii) negative external mechanical work increases with increasing slope in DR; iii) *Vastus Lateralis* muscle oxygenation is higher at maximal intensity in DRSS compared with all other conditions; and iv) knee extensor isometric muscle torque is preserved after maximal LR and UR, but reduced after both DR conditions, despite lower b[La]^−^ in DRSS. All together, these results suggest that the progressive reduction of V̇O_2_ at maximal effort with increasing slope in DR might be related to the metabolic consequences of increased lower limb negative external work (i.e., eccentric muscle actions).

One of the limitations of the present study may lie in the fact that only men were recruited and that it is not possible, at this stage, to state with certainty that these results are transferable to women participants.

### Beyond − 7.5% slope, the subjects, even familiarized to downhill running, do no longer achieve LR V̇O_2peak_

The present physiological and biomechanical responses are in line with a previous investigation, confirming a limitation of V̇O_2peak_ at steep negative slopes during maximal running exercise ^3^. However, our observations extent previous data showing that LR V̇O_2peak_ can still be reached by trained male runners at moderate slope (i.e., − 7.5%). Based on an extrapolation of the V̇O_2_/running velocity relationship measured in DRSS, the participants should have sustained more than 27 km·h^−1^ during 2-min while running at − 15% slope in order to reach LR V̇O_2peak_ (22.4 ± 1.5 km·h^−1^ for 52.5 ± 3.5 ml·min^−1^·kg^−1^ in DRSS vs. 16.4 ± 1.2·km·h^−1^ and 60.1 ± 4.1 ml·min^−1^·kg^−1^ in LR). Although this challenge clearly exceeded our participants’ exercise capacity, they were still able to reach LR V̇O_2peak_ (59.4 ± 5.6 ml·min^−1^·kg^−1^) at moderate slope (− 7.5%) with a vV̇O_2peak_ of 20.9 ± 1.8 km·h^−1^. This is at odds with the data from Liefeldt et al. [23], who reported a diminished V̇O_2max_ in a shallow slope (~ − 5%) in highly-trained subjects. This discrepancy might originate from different familiarization strategies and questions the mechanisms underlying the reduction of V̇O_2peak_ with increasing slope in DR. Whether our group of trained runners may actually have been limited by the mechanical constraints of very high running velocities (i.e., maximal stride length/frequency or insufficient ground contact time to produce the required external mechanical work) remains an open question. To date, DR is considered a model of eccentric muscle action, and share some physiological responses with incremental eccentric cycling where V̇O_2max_ is also markedly reduced [24]. Therefore, the model of DR at moderate slope (− 7.5%) still allows very high levels of oxygen uptake to be reached and suggests an increasing contribution of eccentric muscle actions in the limitation of V̇O_2peak_ occurring at more severe downhill slopes [25].

Conversely to the oxygen uptake limitation observed at maximal effort in DRSS vs DRMS and LR, LR V̇O_2peak_ was reached in both uphill running conditions at exhaustion (Table 1). Nevertheless, there is no consensus on this point. Some authors found a 3 to 6% higher V̇O_2max_ in uphill running in various populations [26–28], whereas our results support most previous studies that reported a similar V̇O_2peak_ between uphill and level running [3, 29–31].

In the present study, O_2_ pulse, that is considered as an indirect surrogate of stroke volume, was higher in UR. This is in line with previous results of Lucas et al. [15] who reported that that peak O_2_ pulse exhibited a notable increase during a maximal incremental uphill running test compared to tests conducted on level ground.

It is noteworthy that, despite a lower V̇O_2peak_ in DRSS compared to the other conditions, HR_peak_ (range: 188–192 bpm) and maximal ventilation (range: 139–152 l·min^−1^) were reached in all five conditions (Fig. 4). The two DR conditions led to progressively lower tidal volumes (2.2 to 2.5 l in DRSS and DRMS respectively vs. 2.8 to 2.9 l in the other conditions) and higher breathing frequencies. Therefore, as previously reported during maximal DR at steep slopes [3] and submaximal eccentric cycling [32], a more superficial ventilation pattern was also observed in DRSS and DRMS. Although the physiological tenets of this result are not presently clear, they might be related to a combination of several factors including higher running speeds mechanical constraints in the lower limbs, exacerbated muscle work for trunk stabilization, altered locomotor/ventilation coupling [3, 33] and/or triggered sympathetic activation pathways [34].

### *Vastus Lateralis* Muscle Oxygenation is Higher During Maximal Downhill Running at Steep Slope

At exhaustion, the attenuated reduction in oxyhaemoglobin from the *Vastus Lateralis* muscle in DRSS versus LR only is in line with other physiological responses as the lower V̇O_2peak_ or the the lower *b*[La]^−^. The tissue O_2_ saturation index was higher in DRSS versus all other conditions, except versus DRMS which was itself higher than LR. Taken together, these results suggest a decrease of the muscle O_2_ extraction measured by NIRS, possibly responsible for the limitation of V̇O_2_ at exhaustion, despite very high running speeds. While LR V̇O_2peak_ could not be reached in DRSS, Δ[HHb] in DRSS correlated inversely to the difference between LR V̇O_2peak_ and DRSS V̇O_2peak_, highlighting that the greater the deoxygenation amplitude in *Vastus Lateralis*, the higher the V̇O_2peak_ values in DRSS. More specifically, the ability to sustain high speeds, influenced by the musculo-nervous system and central factors, appears to be an exercise constraint and should not be considered the sole limiting factor of V̇O_2peak_. Low hemoglobin deoxygenation could result not only from low oxygen extraction, but also from high tissue blood perfusion due to short ground contact times during muscle contractions. The Δ[HHb]:ΔV̇O_2_ ratio in DRSS correlated to the difference between LR V̇O_2peak_ and DRSS V̇O_2peak_, implying that the greater the ratio between deoxygenation amplitude in *Vastus Lateralis* related to the oxygen consumption, the greater the impairment of V̇O_2peak_ between DRSS and LR. Nevertheless, a possible role of muscle oxygenation in the limitation of V̇O_2peak_ observed in DRSS definitely warrants further investigations because V̇O_2_ at maximal intensity was significantly altered between DRSS and DRMS while *Vastus Lateralis* muscle oxygenation parameters remained unaltered in this later condition.

### Maximal Downhill Running at Steep Slopes as a Model of Eccentric Muscle Work

In contrast to DRMS, LR V̇O_2peak_ was not reached in DRSS and this particular condition of severe downhill slope is characterized by reduced propulsive phase (i.e., propulsive parallel force, propulsive impulse, and positive external work), higher braking phase (normal impact force and negative external work) and might have approached some biomechanical limits (i.e., mechanical inability of the subjects to run faster possibly due to a ‘ceiling effect’, since several similar running kinematics values were observed in both DR; Table 2) with imbalance consequences (i.e., higher lateral forces). Taking all these results together, DRSS widely relied on eccentric muscle actions and muscle ability to exert pretty much negative than positive power. Unlike UR, which preferentially requires concentric locomotor muscle actions, DR requires braking muscle actions from the lower limb muscles and is considered mainly as an eccentric exercise modality [1]. Eccentric exercises combine a high level of mechanical muscle tension (i.e., high normal impact force and negative external work) while limiting the associated energy expenditure to generate the necessary braking force within the major extensor muscles of the lower limbs (i.e., higher normal impact force), due to different factors including non ATP-dependent detachment of actin-myosin bridges [35, 36] and/or increased spring-like properties of muscle–tendon units) [37]. Contrary wise, UR relies predominantly on concentric muscle actions (i.e., shortening muscle actions) to move the runner’s body up despite gravity.

Although running speed was 9% higher in DRSS versus DRMS, this increase seems lower than between the other conditions. Moreover, no differences occurred between DRSS and DRMS for the contact/aerial times, and for the stride frequency. Therefore, the higher velocities achieved at maximal effort in DRSS versus DRMS were only due to greater stride lengths. The participants probably reached their physiological limit and were not able to reduce further the contact time. As a consequence, the participants were not able to develop their maximal parallel ground reaction force (compared to the other conditions) possibly limiting V̇O_2peak_. When downhill slope increase, the cyclic motion of the legs also needs to increase and therefore the propulsive force should be produced in a shorter amount of time, reducing the contact time. In sprinting, the peak eccentric torque of the knee flexors is a predominant factor of propulsive force development [38]. It can therefore be hypothesized that the participants were not able to increase peak torque of the knee extensors to generate enough parallel propulsive ground reaction force. Finally, as highlighted by the higher negative lateral external work and total lateral forces in DRSS versus all other conditions, the lateral imbalance was more important in the steepest negative slope, inducing a higher need for global body balance and especially for trunk stabilization, possibly contributing to reach peak/maximal values of ventilation and heart rate in DRSS.

### Global Neuromuscular Fatigue, Delayed-Onset Muscle Soreness and Rate of Perceived Effort

A significant torque loss occurred only after both DR conditions. This global neuromuscular fatigue is typically associated with high-intensity eccentric muscle exercises, as downhill running at steep slopes [33, 39–41]. Even if the global RPE was similar at exhaustion between conditions, the muscle RPE was higher and the breathing RPE lower in DRSS only; further emphasizing the higher mechanical than metabolic load (i.e., negative external mechanical work) at steep DR slope. One could expect that this may also be the case for DRMS, since DOMS values for gluteal, knee extensor, hamstring, and plantar flexor muscles were elevated at least up to 3 days after both DR conditions, although all subjects were fully familiarized. This observation provides important information regarding athletes’ muscle protection to maximal DR and suggest that more frequent, longer duration and/or higher intensity familiarization strategies are required to fully protect lower limb muscles from maximal intensity DR-induced muscle damages.

## Conclusion

This study demonstrates that a group of trained male runners did not achieve a maximal oxygen uptake during maximal incremental running at steep negative slope (− 15%), whereas they were still able to reach LR V̇O_2peak_ at moderate negative slope (− 7.5%). This result occurred despite much higher running velocities in DRSS (> 20% of LR speed at V̇O_2peak_) and the achievement of peak/maximal HR and ventilation at exhaustion. The limitation of V̇O_2peak_ observed in DRSS was concomitant to significant alterations of ground reaction forces: i) reduced propulsive phase (i.e., propulsive parallel force, propulsive impulse and positive external mechanical work), ii) greater braking phase (normal impact force and negative external mechanical work). Additionally, the higher muscle oxygenation (lower deoxygenation) observed in DRSS suggests that oxygen extraction was not maximized in this condition. These observations contribute to a better understanding of the specific physiology and biomechanics of DR, highlighting the effect of slope on putative limiting factors of oxygen uptake in trained male runners performing maximal effort in DR.

## Data Availability

Due to ethical restrictions, the datasets generated for this study are available on request to the corresponding author.

## Abbreviations

*b*[La]^−^: Blood lactate concentration in mmol·l^−^^1^
DOMS: Delayed onset muscle soreness
DR: Downhill running
DRMS: Downhill running in moderate slope
DRSS: Downhill running in steep slope
HHb: Deoxygenated haemoglobin in μM
HR: Heart rate in bpm
HR_peak_: Peak heart rate in bpm
LR: Level running
NIRS: Near-infrared spectroscopy
O_2_Hb: Oxyhaemoglobin in μM
UR: Uphill running
URMS: Uphill running in moderate slope
URSS: Uphill running in steep slope
RPE: Rate of perceived exertion
tHb: Total haemoglobin in μM
TSI: Tissue oxygen saturation index in %
VLR: Vertical loading rate in N·s^−1^·kg^−1^
V̇O_2_: Oxygen consumption in mlO_2_·kg^−1^·min^−1^
V̇O_2max_: Maximal oxygen consumption in mlO_2_·kg^−1^·min^−1^
V̇O_2peak_: Peak oxygen consumption in mlO_2_·kg^−1^·min^−1^
vV̇O_2peak_: Running velocity at peak oxygen consumption in km·h^−1^
W_ext_: External work in J·m^−1^·kg^−1^
W_ext_^−^: Negative external work in J·m^−1^·kg^−1^
W_int_: Internal mechanical work in J·m^−1^·kg^−1^
